# KRAS inhibitors: resistance drivers and combinatorial strategies

**DOI:** 10.1016/j.trecan.2024.11.009

**Published:** 2024-12-27

**Authors:** Tamara Isermann, Christine Sers, Channing J. Der, Bjoern Papke

**Affiliations:** 1 Charité – Universitätsmedizin Berlin, Institute of Pathology, Berlin, Germany; 2 German Cancer Consortium (DKTK), Partner Site Berlin, German Cancer Research Center (DKFZ), Heidelberg, Germany; 3 Lineberger Comprehensive Cancer Center, University of North Carolina at Chapel Hill, Chapel Hill, NC 27599, USA; 4 Department of Pharmacology, University of North Carolina at Chapel Hill, Chapel Hill, NC 27599, USA

## Abstract

In 1982, the RAS genes *HRAS* and *KRAS* were discovered as the first human cancer genes, with *KRAS* later identified as one of the most frequently mutated oncogenes. Yet, it took nearly 40 years to develop clinically effective inhibitors for RAS-mutant cancers. The discovery in 2013 by Shokat and colleagues of a druggable pocket in KRAS paved the way to FDA approval of the first covalently binding KRAS^G12C^ inhibitors, sotorasib and adagrasib, in 2021 and 2022, respectively. However, rather than marking the end of a successful assault on the Mount Everest of cancer research, this landmark only revealed new challenges in RAS drug discovery. In this review, we highlight the progress on defining resistance mechanisms and developing combination treatment strategies to improve patient responses to KRAS therapies.

## The rise of RAS oncogenes as undruggable cancer targets

**Figure 1.**
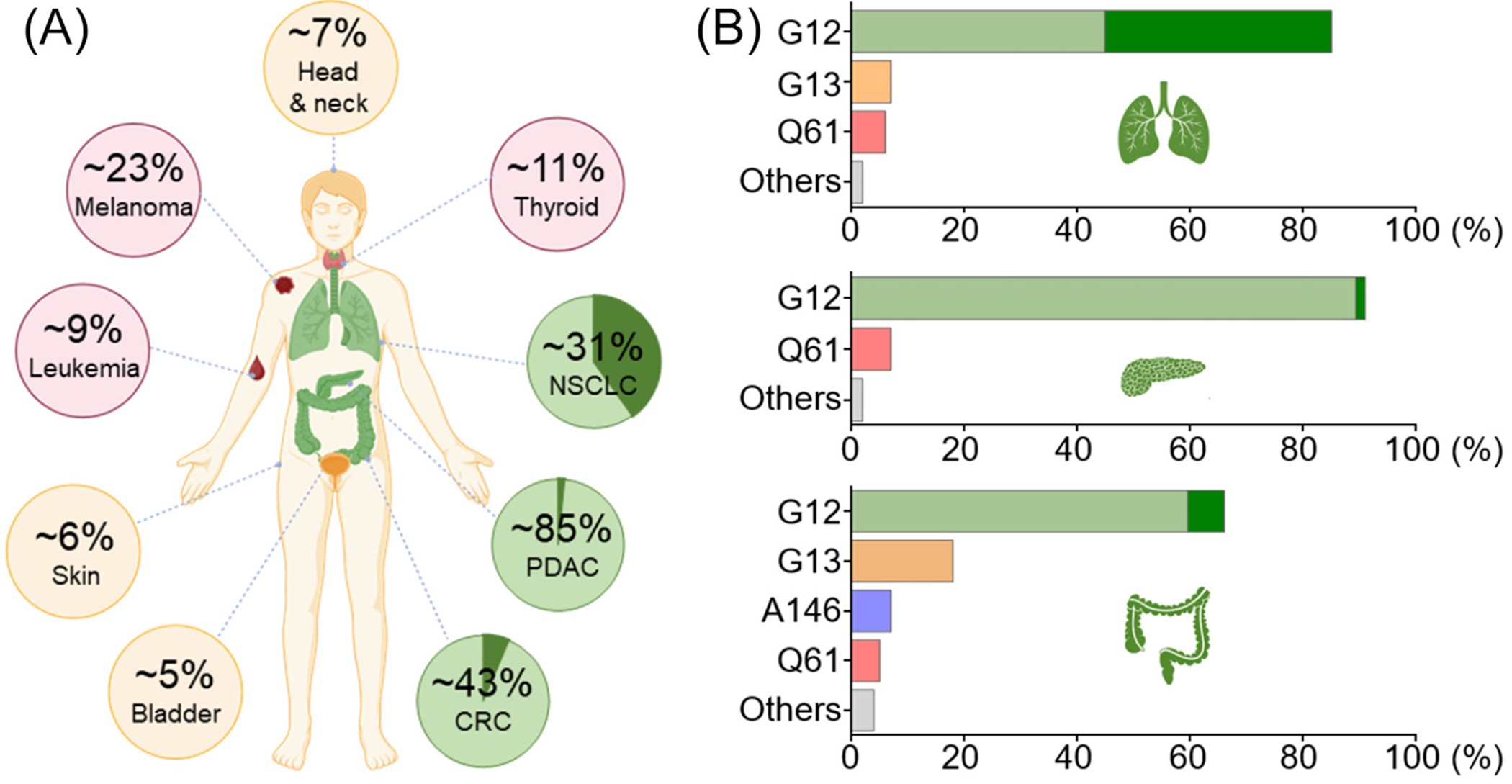
Frequency of RAS mutations in major cancer types. (A) Distribution of *KRAS* (green), *HRAS* (yellow), and *NRAS* (pink) mutation frequencies across the major cancer types. Dark-green sections represent the portion of KRAS^G12C^-targetable cases. (B) Most frequently mutated amino acids (>5%) in KRAS-mutated cancers: top, non-small cell lung cancer (NSCLC); middle, pancreatic ductal adenocarcinoma (PDAC); bottom, colorectal cancer (CRC). Bars represent the percentage of each mutation within the respective cancer entity with dark green sections indicating targetable KRAS^G12C^. Data from cBioPortal^ii^.

The year 1982 marked the beginning of a new era in cancer research, with the discovery of *HRAS* and *KRAS* as the first human cancer genes [1,2]. Since then, the RAS gene family, comprising *HRAS*, *NRAS*, and *KRAS*, has been identified as one of the most important oncogene groups in cancer [3,4]. Among all RAS genes, *KRAS* is the most frequently mutated, accounting for ~80% of all RAS mutations [5,6]. Notably, RAS mutations exhibit significant tissue specificity, with the most common and lethal cancer types, such as colorectal cancer (CRC), pancreatic ductal adenocarcinoma (PDAC), and non-small cell lung cancer (NSCLC), being prominently associated with *KRAS* mutations (43%, 85%, and 31%, respectively) (Figure 1A) [7]. By contrast, *HRAS* mutations are commonly linked to skin cancer (excluding melanoma) (6%), bladder cancer (5%), and head and neck cancer (7%), while *NRAS* mutations are prominent in melanoma (23%), thyroid cancer (11%), and leukemia (9%)^i^ (Figure 1B). RAS proteins are key regulators of oncogenic signaling pathways, including mitogen-activated protein kinase (MAPK) and phosphoinositide 3-kinase (PI3K) pathways. This is further underscored by the high frequency of cancer-driving mutations in RAS downstream effectors (Box 1), including oncoproteins, such as BRAF (8%), AKT1/2 (4%), MEK1/2 (2%), and PIK3CA (13%)^i^, which all result in constitutive protein activity promoting tumor growth and survival. The important role of RAS proteins and RAS downstream effectors as drivers of cancer has led to an intense search for effective RAS inhibitors.

Early on, experimental studies using cell culture and mouse models confirmed mutant RAS as a critical driver of cancer development and progression, further validating it as a favorable cancer target [8,9]. However, the tertiary structures of RAS proteins revealed the lack of deep hydrophobic pockets on the surface, limiting opportunities for the design of potent and selective small-molecule inhibitors [10,11]. In addition, the exceptionally high picomolar affinity of RAS for GTP, together with the high cellular concentration of GTP (~500 μM) renders RAS targeting significantly more challenging compared with kinases, which bind ATP with micromolar affinity and ATP concentrations in the millimolar range [12–14]. This contributed to the longstanding belief that RAS was undruggable [15,16]. Consequently, early strategies aimed at targeting RAS were primarily indirect.

The first and most promising approach involved the development of farnesyltransferase inhibitors (FTIs) to disrupt localization of RAS at the plasma membrane, which is essential for RAS to function in signal transduction [17,18]. FTIs failed dramatically in clinical trials (NCT00100750, NCT00005989, and NCT00005648) due to unexpected RAS isoform differences, where KRAS and NRAS, but not HRAS, could escape the actions of FTIs through alternative prenylation [19]. This failure marks one of the darkest hours in KRAS history. Subsequently, research efforts shifted focus toward targeting KRAS effector signaling, in particular the RAF-MEK-ERK MAPK pathway, which led to the development of multiple inhibitors targeting each level of this cascade. However, MAPK pathway inhibitors have shown limited efficacy in KRAS-driven cancers, primarily due to unexpected dynamic feedback mechanisms and significant toxicity in normal tissues [20,21].

In this review, we summarize the groundbreaking journey from the development of the first KRAS^G12C^ inhibitor to FDA approval of KRAS-targeted therapies nearly 40 years after RAS mutations were first identified as human oncogenes. We discuss the current impact of, and advances in, KRAS inhibitors in clinical settings, address the persistent challenge of therapeutic resistance, and review ongoing clinical evaluations of combination therapies aimed at enhancing sensitivity to KRAS inhibitors or overcoming resistance.

## Development of KRAS^G12C^ inhibitors

The first findings that began to dispel the notion that RAS was an undruggable protein involved the discoveries of Kobe 0065 and SML-8731. Kobe 0065 inhibits the binding of HRAS to RAS-binding domains in effector proteins [22] and SML-8731 was reported to bind to the nucleotide-binding domain of KRAS^G12C^ [23]. However, neither of these small molecules advanced to clinical evaluation.

In 2013, Shokat, Wells, and colleagues reported the seminal findings that finally paved the way to success. They screened for an inhibitor that covalently binds to the cysteine of the glycine-12-cysteine (G12C) substitution of mutant KRAS^G12C^ [24]. Using a small-molecule library of cysteine-reactive compounds (so-called ‘warheads’), they identified several hits, leading to the development of compound 12 [24]. The development of the trailblazer compound 12 was a pivotal moment in KRAS drug design. Structural analyses revealed that this compound bound to a previously unrecognized pocket within the switch II region, designated S-IIP [24,25]. Although compound 12 exhibited low potency, it demonstrated selective inhibition of KRAS^G12C^ mutant cancer cells [24]. These findings not only established that the undruggable KRAS was druggable, but also highlighted that this can occur in a mutant-selective manner, reducing toxicities to healthy tissue harboring wild-type (WT) KRAS.

Additional chemical modification of compound 12 led to the development of ARS-853, which exhibited improved cellular potency *in vitro* [26]. Subsequent further optimization resulted in the development of ARS-1620, with enhanced potency and oral bioavailability *in vivo* [27]. ARS-1620 demonstrated potent antitumor activity in KRAS^G12C^-mutant cell line-derived (CDX) and patient-derived (PDX) xenograft mouse models. A surprising characteristic of compound 12 and subsequent compounds was their selectivity for the inactive (OFF) GDP-bound form of KRAS^G12C^ [28]. At that time, the dogma in the field was that all RAS mutants were created equal, simply frozen in the GTP-bound state and, consequently, expected to be insensitive to an OFF-state selective inhibitor [29,30]. However, it was determined that the KRAS^G12C^ mutant dynamically undergoes GDP-GTP cycling and, thus, exists in a state that is responsive to OFF-selective inhibitors [31,32].

In parallel, several pharma companies initiated their KRAS^G12C^ programs and designed small molecules chemically similar to compound 12 that bound to S-IIP and covalently modified GDP-bound KRAS^G12C^. Sotorasib (AMG510) was the first G12C-targeted inhibitor to enter clinical evaluation [33]. In 2018, the first clinical trial, CodeBreaK 100 (NCT03600883), started to study the clinical safety of sotorasib in patients with advanced KRAS^G12C-^mutant solid tumors that had advanced on standard-of-care chemotherapy or immunotherapy. No dose-limiting toxicities were observed, and a 32.2% confirmed **objective response rate (ORR)** (see Glossary) was revealed. **Disease control (DC)** was observed in 88.1% of patients, with a median **progression-free survival (PFS)** of 6.3 months [34]. In CRC, 7.1% had a confirmed **partial response (PR)**, 73.8% experienced DC, and the PFS was 4.0 months [34]. **Complete response** was observed in 0% of patients, with 8.5% of patients with NSCLC and 23.8% of patients with CRC presenting **progressive disease (PD)**. Based on these positive outcomes, CodeBreaK 100 continued into a Phase 2 trial. Here, 80.6% of patients with NSCLC displayed DC, PFS was 6.8 months, and median overall survival (OS) was 12.5 months [35]. Based on the favorable findings of CodeBreaK 100, sotorasib gained accelerated approval from the FDA in May 2021 for locally advanced or metastatic KRAS^G12C^-mutant NSCLC [36,37]. The outcomes of major clinical trials involving KRAS^G12C^ inhibitors are summarized in Table 1.

To further evaluate the benefits of sotorasib, the Phase 3 CodeBreaK 200 trial (NCT04303780) compared sotorasib with docetaxel in patients with NSCLC [38]. Docetaxel, which is a second-line standard-of-care treatment, displayed modest clinical benefit and substantial toxicity. PFS for patients treated with sotorasib was only 1.1 months longer than for docetaxel, 5.6 months versus 4.5 months, respectively. Disappointingly, sotorasib did not show significant improvement in OS, 10.6 versus 11.3 months, respectively, leading the FDA to refuse full approval in 2024, but with accelerated approval still in place. Suboptimal trial design was cited as one basis for this decision [39].

A second KRAS^G12C^-selective covalent inhibitor, adagrasib (MRTX849), also began clinical evaluation in 2018 (KRYSTAL-1) for patients with advanced and metastatic solid tumors bearing KRAS^G12C^ mutations who had been previously treated with chemotherapy and anti-PD-1/PD-L1 therapy (NCT03785249) [40]. Adagrasib presented good bioavailability *in vivo* and demonstrated effective tumor inhibition in KRAS^G12C^ CDX and PDX tumor models [41]. In Phase 1/2 evaluation, adagrasib demonstrated positive results in NSCLC with an ORR of 42.9%, DC was 86%, PFS was 6.5 months, and OS was 12.6 months [42]. Based on these results, the FDA also granted adagrasib accelerated approval for NSCLC in December 2022 [43].

Both sotorasib and adagrasib monotherapy exhibited significantly lower ORR in CRC compared with NSCLC (Table 1). This divergence with KRAS^G12C^-mutant CRC showing poorer response was reminiscent of earlier findings with a poor response of BRAF-mutant CRC versus melanoma to BRAF-inhibitor therapy [44,45]. Epidermal growth factor receptor (EGFR) activation was identified as a basis for the lower response in CRC, with additional EGFR inhibition together with BRAF inhibitor treatment overcoming this issue [44]. This led to the approval of a combined EGFR-BRAF inhibitor treatment for CRC. As a result, the CodeBreaK 300 trial (NCT05198934) was initiated, which combined sotorasib with the EGFR inhibitor panitumumab in KRAS^G12C^-mutated advanced CRC [46]. Combining sotorasib with panitumumab improved PFS to 5.6 months compared with 2.2 months for the patient group receiving standard of care (trifluridine-tipiracil or regorafenib) [46]. The CodeBreaK 300 trial especially highlighted the benefit of combination therapies for KRAS^G12C^ inhibitors. A similar improvement in ORR (30.2%) was observed with adagrasib in combination with cetuximab [47]. Based on these findings, the FDA granted accelerated approval of this combination for KRAS^G12C^-mutated CRC in June 2024.

## Second-generation KRAS^G12C^ inhibitors

**Figure 2.**
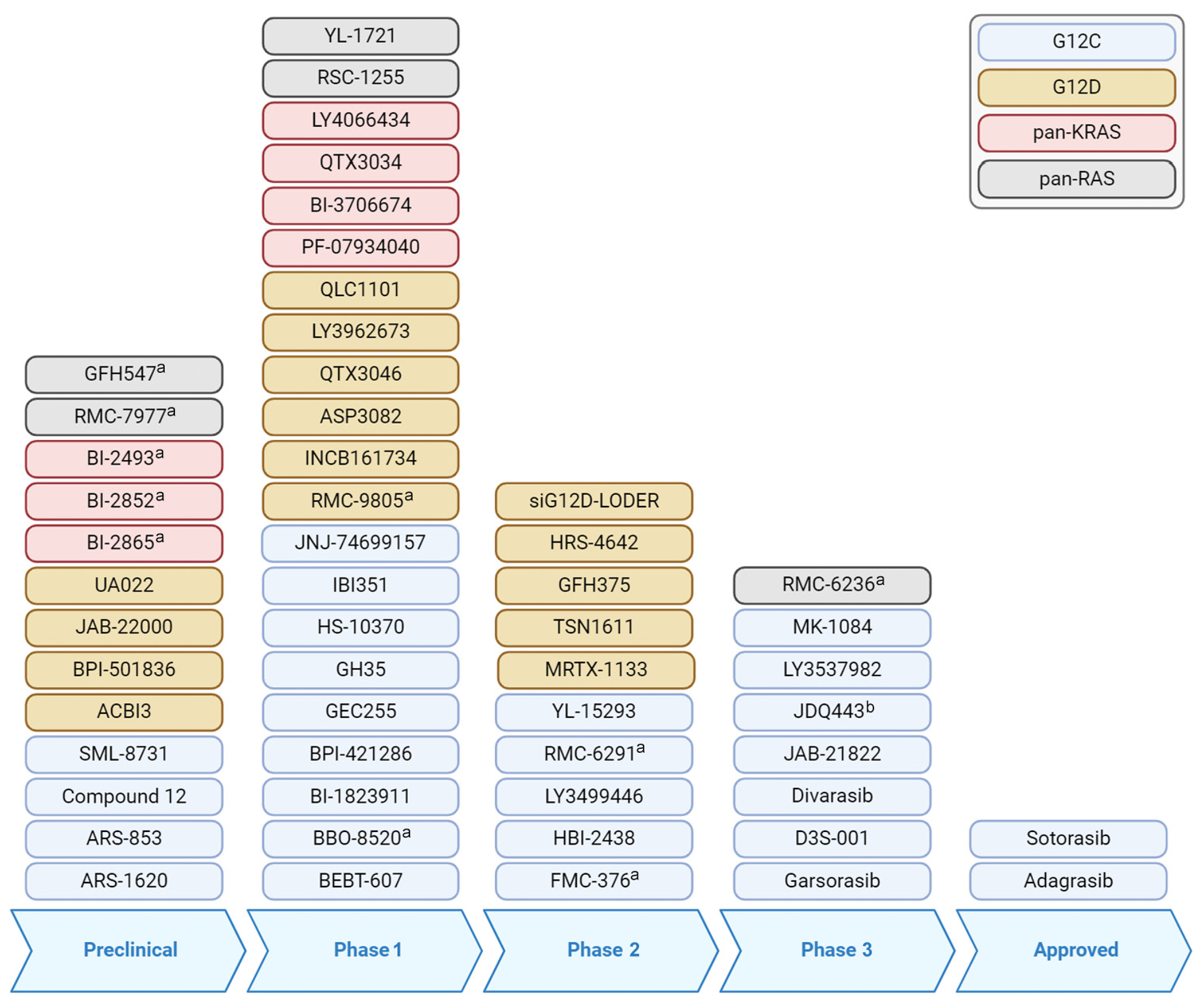
Clinical stages of RAS inhibitors. The clinical stage for each RAS inhibitor is indicated by the arrows below the inhibitors. Light-blue boxes represent KRAS^G12C^ inhibitors, yellow boxes highlight KRAS^G12D^ inhibitors, red boxes mark pan-KRAS inhibitors, and gray boxes display pan-RAS inhibitors. ^a^Indicates GTP-ON state inhibitors; ^b^indicates discontinued inhibitors.

Typically, the initial approvals in a new drug class will not be best in class and, consequently, a multitude of second-generation KRAS^G12C^ inhibitors are under clinical evaluation (Table 2 and Figure 2). Among those, divarasib, a promising KRAS^G12C^ inhibitor, recently entered clinical trials (NCT04449874). Studies demonstrated improved outcomes for patients treated with divarasib compared with sotorasib or adagrasib [48]. Specifically, the ORR of patients treated with divarasib was 53.4% compared with 28.1–43% observed with sotorasib or adagrasib. Additionally, the PFS was extended to 13.1 months in patients with NSCLC, an improvement of 6 months compared with sotorasib or adagrasib treatment [48].

D3S-001, another next-generation GDP-bound KRAS^G12C^ inhibitor, demonstrates faster target engagement compared with first-generation KRAS^G12C^ inhibitors. As a result, D3S-001 effectively depletes GTP-bound KRAS even in the presence of growth factors, such as EGF. D3S-001 has demonstrated enhanced inhibitory effects *in vitro* and is in Phase 1/2 clinical trials (NCT05410145) [49].

While most second-generation inhibitors are mechanistically similar to the original OFF-selective compound 12, two very distinct covalent G12C-selective inhibitors have also entered clinical evaluation. First, BBO-8520 is a covalent inhibitor selectively active on both GDP- and GTP-bound KRAS^G12C^ [50]. Consistent with its ON-state activity, preclinical studies found that BBO-8520 was less susceptible to signaling activities that promote GTP-KRAS^G12C^ formation. BBO-8520 recently entered clinical evaluation for metastatic KRAS^G12C^-mutant tumors (NCT06343402).

Second, RMC-6291 is a **KRAS^G12C^(ON) inhibitor** with a very distinct mode of action compared with S-IIP-targeted inhibitors. RMC-6291 cannot bind directly to KRAS^G12C^ but instead first forms a binary complex with the immunophilin cyclophilin A (CYPA) [51]. This RMC-6291-CYPA complex then forms a tricomplex with GTP-KRAS^G12C^ (RMC-6291 covalently binds to KRAS^G12C^), thereby impairing effector binding and activation by KRAS [51]. RMC-6291 is in Phase 1 clinical trials alone (NCT05462717) and in combination with the multi-RAS inhibitor RMC-6236 or cytotoxic drugs (NCT06128551 and NCT06162221) in KRAS-^G12C^-mutant solid tumors. Additional KRAS^G12C^ inhibitor candidates currently under clinical evaluation include JDQ443 (NCT04699188), BEBT-607 (NCT06117371), and GH35 (NCT05010694).

The field of RAS inhibitors is evolving rapidly, with many new inhibitors in development (Figure 2), leading to an increasingly crowded therapeutic space. As a result, the further development of JDQ443 was recently announced to be discontinued.

## KRAS^G12D^-selective inhibitors

Targeting KRAS^G12C^ has shown significant promise, particularly in NSCLC, which accounts for ~75% of all KRAS^G12C^ cases^ii^. However, non-NSCLC-related KRAS^G12C^ accounts for only ~5% of all KRAS mutations^ii^. Consequently, only a small portion of patients with non-NSCLC-related KRAS^G12C^ would benefit from KRAS^G12C^ inhibitors. Due to the high prevalence of the other KRAS mutations, development of other mutant-selective inhibitors as well as pan-KRAS and pan-RAS inhibitors is ongoing and a subset of these novel RAS inhibitors are being tested in clinical trials (Table 2). In particular, KRAS^G12D^ has emerged as a key area of interest in the development of new inhibitors, given that this mutation represents ~28% of all KRAS mutations^i^ and is the most prevalent mutation in both CRC and PDAC [52–54]. Multiple KRAS^G12D^ inhibitors, including JAB-22000, UA022, and BPI-501836, are being studied in preclinical settings [55–57]. However, MRTX1133, discovered by a structure-based drug design, has pioneered the field of noncovalent KRAS^G12D^ inhibitors [58].

Similar to the FDA-approved KRAS^G12C^ inhibitors, MRTX1133 recognizes KRAS^G12D^ in its GDP-bound state. In addition, MRTX1133 can also bind GTP-bound KRAS, albeit with lower affinity and its selectivity is 700-fold higher for KRAS^G12D^ versus WT KRAS [59]. In preclinical studies, MRTX1133 exhibited potent activity in KRAS^G12D^-mutant pancreatic CDX and PDX mouse models [58,59]. Additionally, upon MRTX1133 treatment, a temporal shift from a basal-like to classical phenotype was observed in both PDAC cell lines and PDX models, which renders the tumor cells more susceptible to chemotherapy [60,61]. MRTX1133 was the first KRAS^G12D^-selective inhibitor to enter clinical evaluation in 2023, with a Phase 1/2 study evaluating MRTX1133 in patients with mutant KRAS^G12D^ with advanced solid tumors (NCT05737706). Similar to the KRAS^G12C^ inhibitors, MRTX1133 is part of an expanding class of small-molecule KRAS^G12D^ inhibitors under clinical evaluation. Other candidates under clinical evaluation include GFH375/VS-7375 (NCT06500676), HRS-4642 (NCT05533463), INCB161734 (NCT06179160), LY3962673 (NCT06586515), TSN1611 (NCT06385925), QLC1101 (NCT06403735), and QTX3046 (NCT06428500). To date, clinical responses have only been reported with HRS-4642 on 18 patients with solid tumors [62]. Six patients experienced target lesion shrinkage, with grade three or less toxicities [62].

Using the CYPA-based tricomplex strategy, RMC-9805 is another KRAS^G12D^ covalent inhibitor but, in contrast to the other KRAS^G12D^ inhibitors, it is selective for the KRAS(ON) state [63]. In seven out of nine PDAC PDX and CDX models and six out of nine NSCLC PDX models, an objective response was obtained following RMC-9805 treatment [63]. Phase 1 clinical evaluation of RMC-9805 revealed a 30% ORR and an 80% DC, with gastrointestinal-related toxicities and rashes for limited periods of time (NCT06040541)^iii^.

Besides the direct small-molecule KRAS^G12D^ inhibitors, two alternative approaches are being tested in clinical trials: proteolysis-targeting chimeras (PROTACs) and an RNAi-based biodegradable implant. PROTACs have recently gained research interest in oncology, because they offer the potential to degrade the target protein, thereby eliminating potential scaffold functions [64]. In general, a broad range of PROTAC-based degraders are under evaluation for the treatment of prostate and breast cancer [65]. Recently, a KRAS PROTAC, namely ACBI3, was demonstrated to target all major oncogenic *KRAS* alleles, effectively degrading KRAS^G12D^ and KRAS^G12V^ in CRC and ovarian leiomyosarcoma xenografts, respectively [66]. Meanwhile, the KRAS^G12D^-targeting PROTAC, ASP3082, has entered Phase 1 clinical trials either as a single treatment or in combination with cetuximab or chemotherapy (NCT05382559) [67,68]. Early results suggest an acceptable safety profile in PDAC, CRC, and NSCLC [69]. Additionally, the RNAi-based biodegradable implant siG12D-LODER is under investigation in a Phase 2 clinical trial in combination with chemotherapy for the treatment of locally advanced pancreatic cancer (NCT01676259). Early results from this study demonstrated that local downmodulation of KRAS is well tolerable and safe [70].

## Pan-KRAS inhibitors

In cancer, RAS genes show a spectrum of mutations beyond G12C and G12D (Figure 1). Therefore, new inhibitors are being developed to target the broad spectrum of RAS mutations. The tool compounds BI-2865 (BI-2493 structural analog) and BI-2852 were designed by eliminating the cysteine-specific covalent warhead (targeting the G12C mutation) and applying structure-based modifications [71,72]. These noncovalent GDP pan-KRAS inhibitors exhibit activity against a range of KRAS mutants, as well as against KRAS WT [71,72]. Since these inhibitors selectively recognize the OFF-state of KRAS, their broad activities suggest that most KRAS mutants, such as G12C, dynamically cycle between GDP- and GTP-bound states. However, these inhibitors lack *in vitro* activity against KRAS mutations with impaired GTP-GDP cycling activity, such as G12R and most Q61 mutations [31]. Nevertheless, these inhibitors resulted in significant growth suppression of KRAS-mutant cancer cell lines and significantly impaired growth of PDX tumors with various KRAS mutations, including G12C, G12D, and G12V [71].

Several pan-KRAS inhibitors are undergoing clinal evaluation. Among these, BI 3706674 is a potent and orally available small molecule that is under Phase 1 evaluation in metastatic KRAS-WT amplified gastric, esophageal, and gastroesophageal junction adenocarcinomas (NCT06056024) [73]. Additional pan-KRAS inhibitors in clinical Phase 1 include PF-07934040 (NCT06447662), LY4066434 (NCT06607185), and QTX3034 (NCT06227377) (Table 2).

## Pan-RAS inhibitors

A mechanism of resistance to KRAS mutant-selective inhibitors is the upregulation of **receptor tyrosine kinase (RTK)** activity, which activates WT KRAS and leads to signaling feedback loops involving other RAS members [74]. The use of pan-RAS inhibitors might counteract this reactivation while also targeting a broader range of RAS mutations [75]. Initially, the concept of pan-RAS inhibition faced significant backlash, because *Kras* had been identified early on as a gene crucial for mouse embryonic development [76,77]. Thus, pan-RAS inhibition was initially thought to have detrimental side effects due to normal tissue toxicity. However, these concerns have not materialized as anticipated. Currently reported pan-RAS inhibitors include GFH547, RSC-1255, YL-17231, RMC-7977, and RMC-6236, with several in clinical trials (Table 2 and Figure 2). The most extensively studied and potentially most advanced are the CYPA-based tricomplex RAS(ON) multiselective inhibitors, RMC-7977 (tool compound) and RMC-6236 (clinical candidate). RMC-7977 targets both mutant RAS and RAS WT in the GTP-bound state [78,79] and has demonstrated potent antitumor activity in RAS-dependent mouse cancer models, including PDAC, CRC, and NSCLC. RMC-7977 induced tumor-selective apoptosis, while the skin and colon of these mice exhibited only a few apoptotic cells [78,79].

The related analog of RMC-7977, RMC-6236, is being tested in clinical trials as monotherapy (NCT05379985), in combination with chemotherapy or immunotherapy (NCT06162221), or in combination with the KRAS^G12C^-selective inhibitor RMC-6291 (NCT06128551). Similar to RMC-7977, preclinical models treated with RMC-6236 tolerated all doses tested and induced strong tumor regression in both PDAC and NSCLC models. In both entities, the median tumor progression-free period was not reached. By contrast, CRC models responded to a lesser extent, with half the preclinical models showing progressive disease and the median tumor regression-free period being reached after 60 days [80]. The results of this study indicate that CRC tumors may require a combination therapy, as has been successfully demonstrated with KRAS-^G12C^ inhibitors and EGFR antibodies in CRC. Nevertheless, Phase 1/2 monotherapy results were reported for 127 patients with KRAS^G12X^ and other RAS-mutant PDAC. The ORR and DC observed were 29% and 91%, respectively in second-line treated patients^iv^. Impressively, two patients exhibited complete responses, one with a KRAS^G12D^-driven PDAC and the other with a KRAS^G12V^-driven NSCLC. These two patients had no evidence of disease after 5 and 8 months of treatment, respectively [80]. The predominant side effects observed in 87% of patients were rashes, 6% of which were grade 3 or higher. Dose modification due to treatment-related adverse events occurred in 35% of patients. However, no events resulted in treatment discontinuation^iv^. In support of these results, recruitment for the RASolute 302 Phase 3 clinical trial is ongoing (NCT06625320). The relatively low PFS of patients with PDAC receiving RMC-6236 either as second or third-line therapy (8.1 and 4.2 months) indicates that, similar to KRAS mutant-selective inhibitors, multi-RAS inhibitors are subject to rapid resistance development.

## The challenge of multiple resistance mechanisms

With the large number of RAS inhibitors being tested in clinical trials, it has become clear that the development of resistance will be a major challenge to their long-term efficacy [81]. Remarkably, multiple resistance mechanisms have been observed in individual patients treated with KRAS^G12C^ inhibitors, ~50% of which may be attributed to genetic alterations [61,81]. Notably, multiple resistance-driving mutations have been identified within individual patients. These resistance mechanisms can be classified as **primary, acquired**, and **adaptive resistance**.

Primary resistance is the underlying cause of non-responders and involves mechanisms that render the tumor unresponsive to KRAS inhibition. This form of resistance occurs in 9–24% of all patients [34]. Although our understanding of mechanisms driving primary resistance to KRAS^G12C^ inhibitors is limited, it will be critical for patient stratification. In a clinicogenomic study of 424 patients with NSCLC treated with sotorasib or adagrasib, co-occurring mutations in *KEAP1*, *SMARCA4*, and *CDKN2A* were independently associated with earlier disease progression and poorer clinical outcome. Notably, the *KRAS^G12C^/KEAP1^MUT^/STK11^MUT or WT^* subgroup exhibited significantly shorter PFS (2.8 months versus 5.3 months) as well as OS (6.3 months versus 10.7 months) compared with the *KRAS^G12C^/KEAP1^WT^/STK11^WT^* subgroup [82]. A meta-analysis of 1224 patients in 18 studies with adagrasib or sotorasib also identified *KEAP1* mutation as a marker of poor response [83]. KEAP1 is a component of an E3 ligase complex that promotes proteasome-dependent degradation of NRF2. Loss of KEAP1 activates NRF2, leading to the regulation of genes that promote an antioxidant protective response. KEAP1 loss has also been identified as a driver of resistance to EGFR inhibition in NSCLC [84,85].

**Figure 3.**
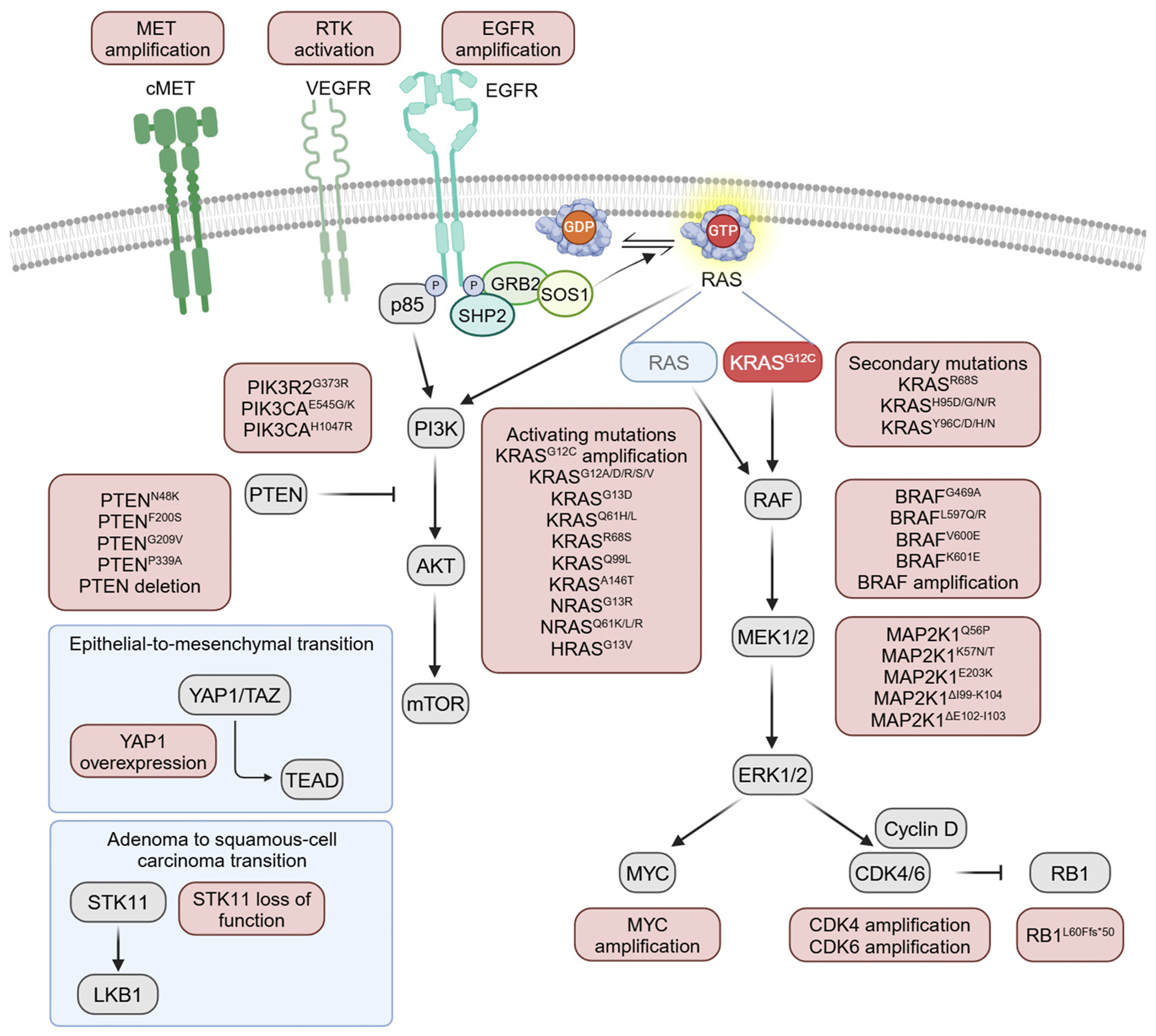
Resistance mechanisms to KRAS^G12C^ inhibition. Red boxes highlight the most frequently observed acquired resistance mechanisms, including KRAS^G12C^ amplifications, activating mutations in KRAS, NRAS, and PIK3CA, as well as secondary mutations in the inhibitor-binding domain of KRAS. Blue boxes indicate adaptive resistance mechanisms, particularly those involving cell state transitions. Abbreviations: EGFR, epidermal growth factor receptor; PI3K, phosphoinositide 3-kinase; RTK, receptor tyrosine kinase; VEGFR, vascular endothelial growth factor receptor.

By contrast, acquired resistance is observed in most patients who initially respond to KRAS^G12C^ inhibition, but then develop resistance after ~4–6 months [81]. This resistance can emerge due to the acquisition of new mutations that impair inhibitor binding to KRAS and, thus, overall efficacy (Figure 3). Within the KRYSTAL-1 trial, 45% of patients who initially had stable disease for a minimum of 12 weeks, developed resistances to adagrasib [81]. Sequence analyses of circulating tumor DNA identified newly acquired *KRAS* mutations with *KRAS^R68S^, KRAS^H95D/G/N/R^*, and *KRAS^Y96C/D/H/N^* being among the most frequently observed resistance mutations [81,86]. These mutations are located within the switch II binding pocket of KRAS, hindering inhibitor binding. In addition, secondary activating *KRAS* mutations were found at lower frequencies, within either the mutant or the WT allele, including G12A/D/R/S/V, G13D, and Q61H/L [81,87]. These newly acquired activating KRAS mutations are not susceptible to KRAS^G12C-^specific inhibitors and, thus, are a potentially additional way to restore oncogenic KRAS activity.

Similarly, *KRAS^G12C^* amplifications have been identified as significant resistance drivers to KRAS^G12C^ inhibitor therapy in both CRC and NSCLC [81,87,88]. *KRAS^G12C^* amplification leads to an increased amount of KRAS protein, which is initially GTP loaded due to the 10:1 GTP:GDP ratio within the cell [89]. Given the slow intrinsic GTPase activity, the steady-state amount of GTP-bound KRAS is elevated, thereby reducing the efficacy of **KRAS^G12C^(OFF) inhibitors**.

Besides *KRAS^G12C^*-altering mutations or amplifications, acquired resistance to KRAS^G12C^ inhibitors can also be driven by the paralog RAS isoforms. Upon KRAS^G12C^ mutation, the main MAPK signaling activity is driven by the mutant KRAS; NRAS and HRAS contributing only minorly. However, patient data indicate that, upon KRAS^G12C^ inhibition, mutations in HRAS or NRAS, for example, NRAS^Q61K/L/R^ and HRAS^G13V^, can restore both MAPK and PI3K-AKT pathway activity [81,90]. The same effect is also reached via loss-of-function mutations in the RAS-GAP neurofibromin 1 (NF1), which have been reported in patients [81]. NF1 loss results in impaired GTPase activity, thereby activating WT RAS to promote reactivation of downstream effector signaling pathways following KRAS inhibition [91].

Apart from RAS proteins, downstream effectors may also acquire resistance-driving gene mutations. Common examples of this are *BRAF^V600E^* mutations, *MAP2K1* mutations, such as *MAP2K1^K57N/T^*, or activating *MAP2K1* in-frame deletions [81]. These alterations result in reactivation of the MAPK pathway independently of KRAS, NRAS, or HRAS, thereby promoting KRAS-^G12C^ inhibitor resistance. Likewise, amplification in *MYC*, comprising a major downstream effector of ERK signaling, was observed in patients, counteracting the inhibitory effect of the KRAS^G12C^ inhibitors [61,87]. Mutations in the PI3K-AKT pathway are also observed, such as loss-of-function mutation of the PTEN tumor suppressor, or PIK3CA gain-of-function mutations, such as PIK3CA^H1047R^, causing hyperactivation of PI3K signaling [90]. Besides mutations in the RAS-RAF-MEK or PI3K-AKT pathways to restore KRAS effector signaling, mutations in cell cycle regulators further contribute to KRAS^G12C^ inhibitor resistance. Loss-of-function mutations in CDKN2A, leading to inactivation of the RB1 tumor suppressor, are found in ~20% of patients with KRAS-mutant NSCLC [92].

Given that RTKs are the major activators of RAS, it is not surprising that mutations or amplifications in RTKs, such as EGFR and MET, are frequently observed as a KRAS^G12C^ inhibitor resistance mechanism [61]. MET is a driver of RAS signaling through SOS-dependent activation of WT RAS. However, amplification of MET is also able to activate PI3K-AKT independently of KRAS, as well as other prosurvival pathways, including the JAK-STAT3 pathway [93,94]. Loss of MET function through siRNA depletion of MET or by treatment with the MET inhibitor crizotinib restored sensitivity to sotorasib treatment both *in vitro* and *in vivo* [75].

Additional mutations that may contribute to resistance against KRAS^G12C^ inhibitor have been identified, including activating mutations in the RET RTK as well as oncogenic fusion proteins, such as EML4-ALK, CCDC6-RET, and NRF1-BRAF [81,90]. However, these mutations have not yet been validated as KRAS^G12C^ inhibitor resistance drivers.

Next to the large number of genetic mechanisms, adaptive nongenetic mechanisms have also been recognized as important contributors to KRAS^G12C^-directed therapy resistance (Figure 3). Adaptive resistance refers to the ability of tumor cells to circumvent therapy through feedback mechanisms or post-translational deregulation of protein levels. A frequently observed adaptive strategy involves reactivation of the MAPK pathway via the activation of upstream signals [74]. WT NRAS and HRAS can become activated as a compensatory mechanism in response to the loss of KRAS^G12C^ activity. This compensatory activation often occurs through the over-expression of RTKs or downmodulation of phosphatases [74]. For example, EGFR overexpression results in increased levels of GTP-bound KRAS^G12C^ and, thus, reduces the amount of inhibitable protein. However, more EGFR also activates WT RAS proteins, such as HRAS and NRAS, which subsequently drive activation of the RAF-MEK-ERK cascade [95]. Similarly, FGFR1 overexpression leads to sustained ERK activation, allowing the cells to overcome inhibition of KRAS^G12C^ [75].

**Epithelial–mesenchymal transition (EMT)**, a phenomenon frequently associated with therapy resistance in various cancer entities [96,97], can promote resistance to KRAS^G12C^ inhibitors. Mesenchymal-like cells promote PI3K activity, despite the presence of KRAS^G12C^ inhibitors, thereby overcoming their inhibitory effect [98,99]. Furthermore, mesenchymal cells show activation of FGFR or AXL to promote resistance, feeding into the HIPPO pathway [100]. This pathway is associated with EMT, with the downstream activator YAP being a driver of EMT. A genome-wide CRISPR/Cas9 screen of KRAS^G12C^-mutant NSCLC cells identified YAP1/TAZ-TEAD activation as an important driver during adagrasib resistance development [101]. These results highlight the potential effect of the HIPPO pathway on EMT and resistance to KRAS inhibitors. Since the TEAD inhibitor, VT3989 is in clinical trials for solid tumors (NCT04665206), the effect of TEAD1 inhibition on adagrasib sensitivity of NSCLC cells was tested preclinically and proven to enhance adagrasib efficiency [102,103].

Not only EMT, but also transition of lung adenocarcinoma to a squamous cell carcinoma phenotype (AST) has been linked to KRAS^G12C^ inhibitor resistance in NSCLC lacking known gene mutations [81,104]. Experimental analyses in genetically engineered mouse models of lung cancer found that loss of function of the *STK11* tumor suppressor gene, which encodes LKB1 serine/threonine kinase, drives AST and resistance to G12C inhibitors. Notably, co-mutation of *STK11* with *KRAS* occurs in 15–30% of lung adenocarcinomas [104].

Given the mechanisms described earlier, it is obvious that single-drug treatments using KRAS inhibitors frequently develop resistance. A key lesson learned from past experiences is that combinatorial therapies are essential for improving patient outcomes. These strategies can help delay or overcome resistance, thereby enhancing the overall efficacy and durability of treatment. The main challenge is the upfront recognition of the most promising combinatorial approaches for individual patients.

## Combinatorial treatments to enhance KRAS^G12C^ inhibitor efficacy

### RAS pathway-related signaling

**Figure 4.**
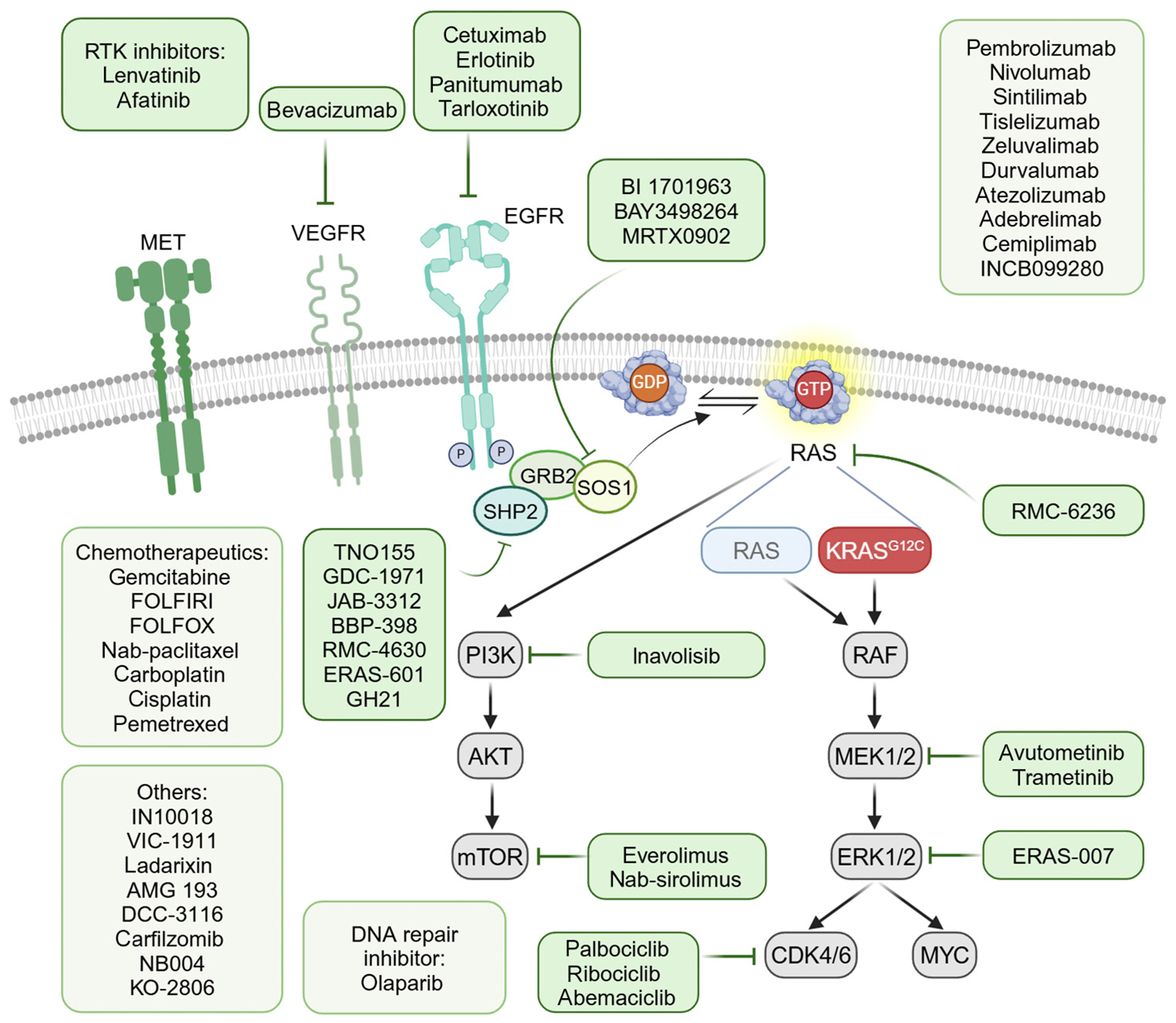
Combinatorial strategies under clinical investigation to enhance KRAS^G12C^ inhibitor efficacy. Green boxes indicate inhibitors targeting up- or downstream effectors of KRAS. Light-green boxes represent inhibitors of KRAS-independent processes, such as DNA-damaging agents or immunotherapeutic inhibitors, including PD-L1/PD-1. Abbreviations: EGFR, epidermal growth factor receptor; PI3K, phosphoinositide 3-kinase; RTK, receptor tyrosine kinase; VEGFR, vascular endothelial growth factor receptor.

Due to the high prevalence of primary and acquired resistance to KRAS^G12C^ inhibitors, research into potential sensitizers and drug combinations is rapidly expanding (Figure 4). Among the most extensively studied combination therapies for KRAS^G12C^ inhibitors are those involving EGFR antibodies, particularly in CRC. This strategy is informed by early RAF inhibitor studies, which were initially ineffective due to feedback-induced upregulation of RTK signaling, in particular EGFR signaling [44,45]. By using **vertical inhibition** of both EGFR and RAF or EGFR and RAS, a more robust suppression of oncogenic signaling is achieved, potentiating the antitumoral effects of the inhibitors [105,106]. Preclinical studies demonstrated that the EGFR inhibitor cetuximab sensitized KRAS^G12C^-mutant CRC CDX and PDX models to KRAS^G12C^ inhibition, resulting in tumor shrinkage [107].

Multiple clinical trials have investigated the efficacy of combining KRAS^G12C^ inhibitors with the EGFR antibodies cetuximab and panitumumab [108] (Table 2). In the Phase 1b KRYSTAL-1 study, the benefit of adagrasib combined with cetuximab was evaluated in CRC, NSCLC, and PDAC. In patients with CRC, the ORR increased from 18.6% with adagrasib alone to 46.4% when treated with the combination [109]. The addition of EGFR antibodies to KRAS^G12C^ inhibitors reduces both mutant and WT GTP-bound RAS [110]. In June 2024, the FDA granted accelerated approval for this specific combination therapy for the treatment of patients with KRAS^G12C^ CRC. Interestingly, the combination of panitumumab with sotorasib is effective not only in patients with KRAS^G12C^-mutated CRC, but also in those with NRAS^G12C^-mutated CRC, as recently described in two patients [111,112]. Both patients exhibited either stable disease or PR for up to 12 weeks of treatment, with the only toxicities being EGFR inhibitor-based rashes. Rubinson *et al*. also demonstrated that sotorasib inhibits all RAS^G12C^ isoforms, with a fivefold higher potency against NRAS^G12C^ than against KRAS^G12C^ or HRAS^G12C^ [112]. However, this ability of sotorasib is not shared with the other FDA-approved KRAS^G12C^ inhibitor adagrasib, which inhibits KRAS^G12C^, but not HRAS^G12C^ or NRAS^G12C^. This specificity is linked to a necessary interaction between the inhibitor and the KRAS residue H95, which is not conserved in HRAS or NRAS [113]. This also accounts for why H95 mutations have been identified in patients who relapsed on adagrasib but not on sotorasib [114].

KRAS^G12C^ inhibitor monotherapy frequently results in loss of multiple ERK-driven negative feedback loops causing reactivation of ERK [115]. Combining MEK inhibitors with KRAS^G12C^ inhibitors presents an approach that could counteract MAPK signaling pathway reactivation. Unfortunately, the combination of KRAS^G12C^ inhibition and MEK inhibition is insufficient to overcome EGFR reactivation [107,116,117]. To address this, a triple vertical combination of MEK and EGFR inhibition together with KRAS^G12C^ inhibition was tested in the CodeBreaK 101 trial [116]. The combination of sotorasib with the MEK inhibitor trametinib and the EGFR antibody panitumumab demonstrated a PR of 20% in patients with NSCLC and 9% in patients with CRC, with a DC of 87% and 82%, respectively^v^.

While EGFR is among the most widely studied RTK targeted in combination with KRAS^G12C^ inhibitors, other RTKs, including VEGF, MET, or AXL, have also been investigated as cotargets. MET amplification drives resistance to EGFR antibodies and also to the KRAS^G12C^ EGFR antibody combination [118,119]. Thus, the inhibition or knockdown of MET in combination with KRAS^G12C^ inhibition resensitizes cells and promotes tumor shrinkage in mice [93]. Another RTK the inhibition of which was shown to sensitize to KRAS^G12C^ inhibitors is AXL. Early on in one of the first CRISPR screens to discover sensitizers for the KRAS^G12C^ inhibitor ARS1620, AXL was identified as a sensitizer in PDAC cells [120]. Additionally, a YAP-AXL axis was described to promote adagrasib tolerance in NSCLC cells, with AXL inhibition sensitizing the cells toward KRAS^G12C^ inhibition [121].

Given the role of multiple RTKs as drivers of resistance, instead of using a complex cocktail of inhibitors targeting specific RTKs, a more effective approach might be to target a common node between RTK signaling and RAS activation. The guanine nucleotide exchange factor SOS1 is crucial in mediating RTK activation of RAS [122]. Thus, inhibiting SOS1 offers a potential pan-RTK-RAS therapeutic strategy. The SOS1 inhibitor BI-3406 in combination with adagrasib resulted in impaired tumor growth, even in adagrasib-resistant models, driven by MRAS and SHOC2 [123]. Similarly, the SOS1:KRAS inhibitor, BI 1701963, in combination with the KRAS^G12C^ inhibitor BI 1823911, resulted in tumor regression in NSCLC CDX and entered clinical trials in 2021 (NCT04973163) [124]. Additionally, a SOS1 PROTAC, based on the SOS1 inhibitor BI-3406, combined with sotorasib synergistically reduced lung tumor volume in mice [125]. This approach inhibits oncogenic KRAS through the KRAS^G12C^ inhibitor, while additional SOS1 degradation prevents RTK-based activation of WT KRAS, NRAS, and HRAS and, therefore, prevents them from compensating for the loss of KRAS^G12C^ function.

Another approach to uncouple RTKs from RAS is to target the SHP2 phosphatase. SHP2 functions downstream of RTKs to promote RAS activation through regulation of SOS1 activity [126,127]. Consequently, SHP2 inhibitors can act as pan-RTK inhibitors and prevent RTK signaling-induced RAS activation. This leads to accumulation of KRAS in the GDP-bound state, allowing for a higher proportion of KRAS^G12C^ being targeted by KRAS^G12C^(OFF) inhibitors. Combinations of SHP2 and MAPK inhibitors have already indicated synergistic effects [128–130]. Additionally, various *in vitro* studies have proven SHP2 inhibitors to potently and sustainably suppress the RAS pathway upon KRAS^G12C^ inhibitor treatment [105,131]. As a result, multiple SHP2 inhibitors are under clinical evaluation in conjunction with KRAS^G12C^ inhibitors. Initial results from the KontRASt-01 trial (NCT04699188), combining the KRAS^G12C^ inhibitor JDQ443 with the SHP2 inhibitor TNO155, were released at the 2023 IASLC World Conference on Lung Cancer. DC rates of 67% were documented for patients pretreated with a KRAS^G12C^ inhibitor, with an ORR of 33%, which lasted more than 6 months [132]. While SHP2 inhibitors demonstrate strong antitumor effects, particularly in preclinical models, they encounter tolerability issues in clinical settings, necessitating optimized dosing schedules to minimize adverse events [133].

To overcome feedback loops driven by activated WT HRAS, NRAS, and KRAS, multi-RAS inhibitors offer a further alternative to block RTK-induced RAS-MAPK signaling. Early Phase 1 clinical trials of these inhibitors reported PR in patients with PDAC and NSCLC [134]. As a result, ongoing clinical trials are exploring the combination of the multi-RAS inhibitor, RMC-6236, with the KRAS^G12C^ inhibitor RMC-6291 (NCT06128551).

Another approach to prevent activation of WT HRAS by RTK feedback loops circles back to the previously described application of FTIs. A recent study demonstrated the benefit of combining sotorasib with the FTI, tipifarnib, in NSCLC, PDAC, and CRC [135]. While oncogenic KRAS^G12C^ signaling is inhibited by sotorasib, the alternative RAS isoforms can compensate for this inhibition. FTIs, which impair HRAS signaling, resulted in enhanced efficacy of the KRAS^G12C^ inhibitor [135]. Here, the combination might also benefit from the broad activity of FTIs on all farnesylated proteins rather than only on HRAS. One of the best known farnesylated proteins also targeted by FTIs is the small GTPase RHEB, which is involved in mTOR signaling, suggesting that PIK3CA/ mTOR signaling is also involved in therapy response.

With KRAS-MAPK signaling being a key driver of the G1–S phase cell cycle transition, targeting of cell cycle-controlling proteins presents an additional vulnerability to improve KRAS inhibition and to address cell cycle-induced drug resistance. Therefore, inhibiting cell cycle regulators, particularly those driving the G1–S phase transition, is a promising cotarget strategy. The cyclin-dependent kinases 4 and 6 (CDK4/6) have a major role in promoting the G1–S phase transition, with their inhibition stalling the cell cycle in G0/G1 [136]. The CDK4/6 inhibitor palbociclib enhanced the efficacy of KRAS^G12C^ inhibitors in cell lines and mouse models [41,120,137]. Currently, five clinical trials are under way in which clinically approved CDK4/6 inhibitors are combined with KRAS^G12C^ inhibitors.

Another cell cycle kinase found to improve KRAS^G12C^ inhibition is Aurora kinase A (AURKA). Combining AURKA inhibition or knockout with KRAS^G12C^ inhibition effectively reduced KRAS reactivation and exerted an antiproliferative effect on cancer cells. Furthermore, AURKA inhibition stimulated KRAS^G12C^ inhibitor-induced quiescence, resulting in numerous cells remaining in the G0 state [138]. Inhibitors of AURKA in combination with sotorasib were also studied in a clinical trial (NCT05374538), although this trial was terminated due to sponsor decisions.

Interestingly, while G0 cell cycle arrest is induced by inhibitors of the RAS-MAPK pathway or cell cycle inhibitors, it can also be triggered by the withdrawal of the KRAS^G12C^ inhibitor in KRAS^G12C^-amplified resistant cells [88]. In this context, drug removal induces a signaling shock, resulting in oncogene-induced senescence. The senescent phenotype of the cancer cells presents a novel vulnerability that could be explored using senolytic drugs [88].

### Immuno-related combination therapies

Immunotherapy is a rapidly advancing field of research, with numerous clinical trials investigating the benefits of anti-PD-1/PD-L1 immune checkpoint therapy in cancer. T cell activity is promoted through the inhibition of either PD-1 or PD-L1, mediating tumor cell death [139–141]. Multiple PD-1/PD-L1-targeting drugs, such as pembrolizumab and nivolumab, have received FDA approval within the past few years for the treatment of NSCLC and other types of cancer [142]. This approach has expanded into combination therapies involving immunotherapy together with KRAS^G12C^ inhibitors in NSCLC and CRC. Preclinical evaluation in a KRAS^G12C^-mutant, immune-competent mouse CRC CDX found that sotorasib alone caused short-term tumor regression followed by tumor regrowth [33]. However, when combined with anti-PD-L1 treatment, complete tumor regression was detected, which persisted for over 2 months after treatment ending. Currently, there are 24 clinical trials combining anti-PD-1/PD-L1 therapy with KRAS^G12C^ inhibitors, the most advanced being the KRYSTAL-7 trial, a Phase 2/3 trial, combining adagrasib with the anti-PD-1 monoclonal antibody pembrolizumab (NCT04613596). No results of the trial have been posted so far.

Given that *PD-L1* mRNA is stabilized by oncogenic RAS signaling and, subsequently, PD-L1 expression is also regulated, combining inhibitors for the two targets appears promising [143]. To further improve this combination, several *in vitro* studies highlighted the benefit of combining KRAS^G12C^ inhibitors not only with anti-PD-1, but also with SHP2 inhibitors. This triple combination is thought to induce antitumor immunity and remodel the tumor microenvironment, potentiating the effects of KRAS^G12C^ inhibition, especially in PDAC [131,144]. Currently, one clinical trial is focusing on these *in vitro* findings, combining JDQ443 with TNO155 and tislelizumab (NCT04699188). No results have been posted so far.

Additional treatments of mutant-KRAS tumors exploiting the immune system involve neoantigens derived from KRAS^G12C^ protein fragments covalently bound to KRAS^G12C^ inhibitors. Once presented on the surface of cancer cells via MHC I complexes, these neoantigens allow bispecific T cell engagers to mediate the interaction between the neoantigen and T cells. This interaction triggers a cytotoxic T cell response, leading to the selective destruction of cancer cells [145,146]. This approach has the advantage that all cancer cells that still harbor the KRAS^G12C^ mutation and are inhibited by the KRAS^G12C^ inhibitor will be targeted by the cytotoxic T cells, even if these cells have developed resistance through mechanisms such as downstream mutations or *MYC* amplification.

### Chemotherapy-related combination therapies

Given that chemotherapy remains the standard of care, it is no surprise that 17 clinical trials are evaluating the combination of chemotherapy with KRAS^G12C^ inhibitors. Preclinical studies provide support for these trials, demonstrating that sotorasib combined with standard-of- care carboplatin resulted in a significantly greater reduction in tumor growth compared with single-drug treatment in a KRAS^G12C^-mutant NSCLC CDX mouse model [33]. Similarly, while treatment with MRTX1133 or standard-of-care gemcitabine alone blocked the metastatic growth in a KRAS^G12D^-mutant PDAC mouse model for only 2 weeks, the combination resulted in prolonged tumor regression [60,61]. Whether the observed effects are due to synergy between KRAS^G12C^ inhibitors and chemotherapeutics or to chemotherapy targeting rapidly replicating cells, including those resistant to KRAS^G12C^ inhibitors, remains an open question.

## Concluding remarks and future perspectives

With the successful approval of two KRAS^G12C^-selective inhibitors, and with numerous inhibitors targeting other KRAS mutations, the perception that KRAS is undruggable can now be put to rest. While most inhibitors under clinical evaluation are mechanistically similar to the two approved mutant-selective OFF allosteric inhibitors, some also present mechanistically distinct approaches, thereby increasing the variety of effective options. Although conventional wisdom is that mutant-selective inhibitors will be superior to inhibitors active on both the WT and mutant KRAS proteins and not be limited by normal tissue toxicity, the unanticipated clinical efficacy of the multi-RAS inhibitor RMC-6236 is challenging this perception.

Moving forward, the most significant challenge for the continued development of KRAS inhibitors is overcoming resistance, which limits the depth and duration of response. It is now generally accepted that the long-term effectiveness of KRAS inhibitors will not be achieved through monotherapy, but rather through combination therapy. While our knowledge of resistance mechanisms remains limited, emerging evidence suggests that these mechanisms will be diverse and complex, varying both within and between different patients with the same cancer type. Thus, establishing effective combinations remains a daunting challenge. Further research is needed, particularly in the fields of patient stratification, combinational approaches, pharmacology, and the understanding of resistance development, to ensure not only improvements in the quality of life of these patients, but also prolonged survival (see Outstanding questions).

After nearly four decades of setbacks, the successful development of clinically effective KRAS^G12C^-selective inhibitors marks a significant milestone in cancer drug discovery. This achievement signals the beginning of an exciting new era of basic and translational RAS research. Previous anti-RAS efforts have been limited by our incomplete understanding of RAS function and future efforts will surely reveal that RAS has many more secrets to unlock. One thing is certain, the future holds promising therapeutic options for patients with RAS-mutant cancers.

## Figures and Tables

**Table 1.** Outcome of FDA-approved KRAS^G12C^ inhibitor clinical trials

Clinical trial	Phase	Cancer entity	Clinical trial number	Drug(s)	ORR (%)	DC (%)	PFS (months)	OS (months)	Refs
CodeBreaK 100	1	NSCLC	NCT03600883	Sotorasib	32.0	88.1	6.3	N/A	[34,37]
		CRC			N/A^a^	73.8	4.0	N/A	[34]
	2	NSCLC			37.1	80.6	6.8	12.5	[37]
CodeBreaK 101	1/2	NSCLC	NCT04185883	Sotorasib + trametinib + panitumumab	N/A	87.0	N/A	N/A	v
		CRC		Sotorasib + trametinib + panitumumab	N/A	82.0	N/A	N/A	v
		CRC		Sotorasib + panitumumab	30.0	92.5	5.7	15.2	[108]
		NSCLC		Sotorasib + carboplatin + pemetrexed	65.0	100.0	N/A	N/A	[148]
CodeBreaK 200	3	NSCLC	NCT04303780	Sotorasib versus docetaxel	28.1 versus 13.2	82.5 versus 60.3	5.6 versus 4.5	10.6 versus 11.3	[37,38]
CodeBreaK 300	3	CRC	NCT05198934	Sotorasib + panitumumab	30.2	71.7	5.6	N/A	[46,149]
KRYSTAL-1	1/2	Solid tumor	NCT03785249	Adagrasib	35.1	86.0	7.4	14.0	[40]
		CRC		Adagrasib + cetuximab	46.0	100	6.9	13.4	[109]
		NSCLC		Adagrasib	42.9	86.0	6.5	12.6	[37,42]
		PDAC		Adagrasib	33.3	81.0	5.4	8.0	[40]
RAMP 203	1/2	NSCLC	NCT05074810	Sotorasib + avutometinib	25.0	N/A	N/A	N/A	vi

a Abbreviation: N/A, not applicable.

**Table 2.** Single and combination therapies of current RAS inhibitors in clinical trials

Inhibitor	Cotarget	Combination	Phase	Tumor type	Clinical trial no^b^.
Adagrasib	N/A^a^	N/A	1	Solid tumor	NCT05263986
				Pancreatic	NCT05634525
	EGFR + topoisomerase	Cetuximab + irinotecan	1	CRC	NCT05722327
	SOS1	BI 1701963	1^d^	Solid tumor	NCT04975256
	PD-L1	Durvalumab	1^c^	Lung and gastrointestinal cancer	NCT05848843
	CDK4/6	Palbociclib	1	Solid tumor	NCT05178888
	PARP	Olaparib	1	Solid tumor	NCT06130254
	N/A	N/A	1/2	Solid tumor, NSCLC	NCT05840510
	mTOR	Nab-sirolimus			
	N/A	N/A	1/2	Solid tumor	NCT03785249
	PD-1	Pembrolizumab		NSCLC	
	EGFR	Cetuximab		CRC, NSCLC, PDAC	
	RTK	Afatinib		NSCLC	
	SHP2	TNO155	1/2	Solid tumor	NCT04330664
	RAF/MEK	Avutometinib	1/2	NSCLC	NCT05375994
	N/A	N/A	2	NSCLC	NCT05673187
					NCT05853575
	N/A	N/A	2/3	NSCLC	NCT04613596
	PD-1	Pembrolizumab			
	N/A	N/A	2	NSCLC	NCT05472623
	PD-1	Nivolumab			
		Pembrolizumab	2	NSCLC	NCT05609578
	PD-1 + chemotherapy	Pembrolizumab + pemetrexed			
		Pembrolizumab + pemetrexed + cisplatin/carboplatin			
	EGFR	Cetuximab versus mFOLFOX6/FOLFIRI	3	CRC	NCT04793958
	Chemotherapy	Versus docetaxel	3	NSCLC	NCT04685135
	PD-L1	INCB099280	1	Solid tumor	NCT06039384
	Radiation therapy	Stereotactic radiosurgery	2	NSCLC	NCT06248606
	EGFR + PD-1/PD-L1	Cetuximab + cemiplimab	1/2	CRC	NCT06412198
	SOS1	MRTX0902	1/2	Solid tumor	NCT05578092
	Farnesyl transferase	KO-2806	1	Solid tumor	NCT06026410
BBO-8520	N/A	N/A	1	NSCLC, CRC	NCT06343402
BEBT-607	N/A	N/A	1	Solid tumor	NCT06117371
BI-1823911	N/A	N/A	1	Multiple	NCT04973163
	SOS1	BI 1701963			
BPI-421286	N/A	N/A	1	Solid tumors	NCT05315180
D-1553 (garsorasib)	N/A	N/A	1/2^d^	NSCLC	NCT05383898
			3	NSCLC	NCT06300177
	N/A	N/A	1/2	Solid tumors	NCT04585035
	PD-1	Pembrolizumab			
	EGFR	Cetuximab			
	Immunotherapy	Name not stated	1/2^c^	NSCLC	NCT05492045
	FAK	IN10018	1/2	Solid tumor	NCT06166836
					NCT05379946
	SHP2	GH21	1/2	Solid tumor	NCT06435455
D3S-001	N/A	N/A	1/2	Solid tumor	NCT05410145
	PD-1	Pembrolizumab			
	chemotherapy	Cisplatin/carboplatin + pemetrexed			
Divarasib	N/A	N/A	2	NSCLC	NCT04302025
			3	NSCLC	NCT06497556
			1	CRC	NCT04929223
			2/3	NSCLC	NCT03178552
	N/A	N/A	1	Solid tumor	NCT04449874
	PD-L1	Atezolizumab			
	EGFR	Cetuximab			
	VEGF	Bevacizumab			
	EGFR	Erlotinib			
	SHP2	GDC-1971			
	PI3Kα	Inavolisib			
	PD-1 + chemotherapy	Pembrolizumab + carboplatin/cisplatin + pemetrexed	1/2	NSCLC	NCT05789082
FMC-376	N/A	N/A	1/2	Solid tumor	NCT06244771
GEC255	N/A	N/A	1	Solid tumor	NCT05768321
GH35	N/A	N/A	1	Solid tumor	NCT05010694
HBI-2438	N/A	N/A	1	Solid tumor	NCT05485974
HS-10370	N/A	N/A	1/2	Solid tumor	NCT05367778
	N/A	N/A	1	Solid tumor	NCT06594874
	PD-L1	Adebrelimab ± pemetrexed, platinum			
IBI351	N/A	N/A	1	CRC	NCT05497336
	EGFR	Cetuximab			
	N/A	N/A	1	NSCLC	NCT05504278
	PD-1	Sintilimab			
	PD-1 + chemotherapy	Sintilimab + pemetrexed			
	PD-1 + chemotherapy	Sintilimab + pemetrexed + cis-platinum/carboplatin			
JAB-21822	N/A	N/A	1/2	NSCLC	NCT05276726
				Solid tumor	NCT05009329
			2	NSCLC	NCT06563999
	N/A	N/A	1/2	Solid tumor	NCT05002270
	EGFR	Cetuximab			
	EGFR	Cetuximab	1/2	CRC, small intestine cancer, appendiceal cancer	NCT05194995
	SHP2	JAB-3312	1/2	Solid tumor	NCT05288205
	N/A	N/A	2	Pancreatic	NCT06008288
	SHP2	JAB-3312	3	NSCLC	NCT06416410
JDQ443	N/A	N/A	1/2	Solid tumor	NCT04699188
	SHP2	TNO155			
	PD-1	Tislelizumab			
	SHP2 + PD-1	TNO155 + tislelizumab			
	MEK	Trametinib	1/2	Solid tumor	NCT05358249
	CDK4/6	Ribociclib			
	EGFR	Cetuximab			
	N/A	N/A	2	NSCLC	NCT05445843
					NCT05714891
			2^c^	NSCLC	NCT05999357
	Chemotherapy	Versus docetaxel	3	NSCLC	NCT05132075
JNJ-74699157	N/A	N/A	1^d^	Solid tumor	NCT04006301
LY3499446	N/A	N/A	1/2^c^	Solid tumor	NCT04165031
	CDK4/6	Abemaciclib			
	EGFR	Erlotinib			
		Cetuximab			
LY3537982	N/A	N/A	1/2	Solid tumor	NCT04956640
	PD-1	Pembrolizumab			
	EGFR	Cetuximab			
	Chemotherapy	Pemetrexed + cisplatin/carboplatin			
	PD-1	Pembrolizumab	3	NSCLC	NCT06119581
	PD-1 + chemotherapy	Pembrolizumab + pemetrexed + cisplatin/carboplatin			
	N/A	N/A	1	Solid tumor	NCT06235983
MK-1084	N/A	N/A	1	Solid tumor	NCT05067283
	PD-1	Pembrolizumab			
	PD-1 + chemotherapy	Pembrolizumab + pemetrexed + carboplatin			
	EGFR	Cetuximab			
	EGFR + chemotherapy	Cetuximab + FOLFOX			
	Unknown	MK-0472	1	Solid tumor	NCT05853367
	PD-1	Pembrolizumab	3	NSCLC	NCT06345729
RMC-6291	N/A	N/A	1	Solid tumors	NCT05462717
	RAS(ON)	RMC-6236	1	Solid tumor	NCT06128551
	PD-1 ± chemotherapy	Pembrolizumab ± cisplatin, carboplatin, pemetrexed	1/2	NSCLC	NCT06162221
Sotorasib	SHP2	BBP-398	1	Solid tumor	NCT05480865
	AURKA	VIC-1911	1^c^	NSCLC	NCT05374538
	Radiation therapy	Stereotactic body radiation therapy	1	NSCLC	NCT06127940
	CXCR1/CXCR2	Ladarixin	1	NSCLC	NCT05815173
	PIM	NB004	1	Solid tumor	NCT05036291
	SOS1	BAY3498264	1	Solid tumor	NCT06659341
	Proteasome	Carfilzomib	1	NSCLC	NCT06249282
	ERK	ERAS-007	1^d^	NSCLC	NCT04959981
	SHP2	ERAS-601			
	N/A	N/A	1	solid tumor	NCT04380753
	PRMT5	AMG 193	1	Thoracic tumor	NCT06333951
	ULK1/2	DCC-3116	1/2	Solid tumor	NCT04892017
	SHP2	JAB-3312	1/2	Solid tumor	NCT04720976
	N/A	N/A	1/2	Solid tumor	NCT04092673
	VEGF	Bevacizumab	1/2^c^	NSCLC	NCT05180422
	N/A	N/A	1/2	Solid tumors	NCT04185883
	MEK + EGFR	Trametinib + panitumumab			
	EGFR ± chemotherapy	Panitumumab ± FOLFIRI			
	VEGF ± chemotherapy	Bevacizumab ± FOLFIRI/FOLFOX			
	SHP2	RMC-4630			
		TNO155			
	SOS1	BI 1701963			
	RTK	Afatinib			
	PD-L1	Atezolizumab			
	PD-1 + chemotherapy	Pembrolizumab + carboplatin, pemetrexed, docetaxel, paclitaxel			
	PD-1	Pembrolizumab			
		Zeluvalimab			
	CDK4/6	Palbociclib			
	mTOR	Everolimus			
	N/A	N/A	1/2	Solid tumors	NCT03600883
	PD-1/L-1	Name not stated			
	EGFR/HER2	Tarloxotinib	1/2^c^	NSCLC	NCT05313009
	RAF/MEK	Avutometinib	1/2	NSCLC	NCT05074810
	Chemotherapy	FOLFIRI/gemcitabine + nab-paclitaxel	1/2^c^	Pancreatic cancer	NCT05251038
	N/A	N/A	2	NSCLC	NCT05631249
					NCT06333678
					NCT04933695
					NCT05398094
					NCT05311709
					NCT05451056
					NCT04625647
			2^c^	NSCLC	NCT05400577
			2	NSCLC	NCT06582771
	EGFR	Panitumumab	2	Solid tumor	NCT05638295
					NCT05993455
	CXCR1/CXCR2	Ladarixin	2^c^	NSCLC	NCT05815186
			1	NSCLC	NCT05815173
	SHP2	RMC-4630 (vicoprotafib)	2	NSCLC	NCT05054725
	RTK	Lenvatinib	2^c^	NSCLC	NCT06068153
	Chemotherapy	Cisplatin/carboplatin + pemetrexed	2	NSCLC	NCT05118854
	EGFR + chemotherapy	Panitumumab + FOLFIRI	3	CRC	NCT06252649
	EGFR	Panitumumab	3	Not specified	NCT05198934
	Chemotherapy	Carboplatin + pemetrexed	3	NSCLC	NCT05920356
		Versus docetaxel	3	NSCLC	NCT04303780
YL-15293	N/A	N/A	1/2	Solid tumor	NCT05119933
					NCT05173805
KRAS^G12D^					
MRTX-1133	N/A	N/A	1/2	Solid tumor	NCT05737706
HRS-4642	N/A	N/A	1	Solid tumor	NCT05533463
	Antibody–drug conjugate	SHR-A1921	1/2	Solid tumor	NCT06520488
		SHR-A1904			
	Chemotherapy	Gemcitabine + paclitaxel			
	PD-L1	Adebrelimab	1/2	PDAC	NCT06427239
	PD-L1 ± chemotherapy	Adebrelimab ± pemetrexed, cisplatin, carboplatin	1/2	Solid tumor	NCT06385678
	Antibody–drug conjugate	SHR-A1921			
	PD-L1	Adebrelimab	2	Biliary tract tumor	NCT06620848
	Chemotherapy	Gemcitabine + paclitaxel	2	PDAC	NCT06587061
	Antibody–drug conjugate	SHR-A2102	2	PDAC	NCT06547736
		SHR-A1904			
		SHR-A1811			
RMC-9805	N/A	N/A	1	Solid tumor	NCT06040541
	Pan-RAS	RMC-6236			
INCB161734	N/A	N/A	1	Solid tumor	NCT06179160
	EGFR	Cetuximab			
	PD-1	Retifanlimab			
ASP3082	N/A	N/A	1	Solid tumor	NCT05382559
	EGFR	Cetuximab			
	Chemotherapy	FOLFIRINOX			
		Nab-paclitaxel + gemcitabine			
GFH375	N/A	N/A	1/2	Solid tumor	NCT06500676
QTX3046	N/A	N/A	1	Solid tumor	NCT06428500
	EGFR	Cetuximab			
LY3962673	N/A	N/A	1	PDAC, NSCLC, CRC	NCT06586515
	Chemotherapy	Gemcitabine, 5-FU paclitaxel, oxaliplatin, leucovorin, irinotecan			
TSN1611	N/A	N/A	1/2	Solid tumor	NCT06385925
QLC1101	N/A	N/A	1	Solid tumor	NCT06403735
pan-KRAS					
BI-3706674	N/A	N/A	1	Stomach, esophagus	NCT06056024
PF-07934040	N/A	N/A	1	Solid tumor	NCT06447662
	Chemotherapy	Gemcitabine/Nab-paclitaxel/FOLFOX/pemetrexed/cisplatin/carboplatin			
	PD-1	Pembrolizumab			
	EGFR	Cetuximab			
QTX3034	N/A	N/A	1	Solid tumor	NCT06227377
	EGFR	Cetuximab			
LY4066434	N/A	N/A	1	Solid tumor	NCT06607185
	EGFR	Cetuximab			
	Chemotherapy	Nab-paclitaxel			
		Gemcitabine			
		Oxaliplatin			
		Leucovorin			
		Irinotecan			
		5-FU			
		Carboplatin			
		Cisplatin			
		Pemetrexed			
	PD-1	Pembrolizumab			
Pan-RAS					
RSC-1255	N/A	N/A	1	Solid tumor	NCT04678648
RMC-6236	N/A	N/A	1	Solid tumor	NCT05379985
	PD-1 ± chemotherapy	Pembrolizumab ± pemetrexed + cisplatin/carboplatin	1/2	NSCLC	NCT06162221
	EGFR ± chemotherapy	Cetuximab ± mFOLFOX6	1/2	Solid tumor	NCT06445062
	Chemotherapy	Gemcitabine + nab-paclitaxel			
		5-FU			
	KRAS^G12D^	RMC-9805	1	Solid tumor	NCT06040541
	KRAS^G12C^	RMC-6291	1	Solid tumor	NCT06128551
	N/A	N/A	3	PDAC	NCT06625320
YL-17231	N/A	N/A	1	Solid tumor	NCT06096974
					NCT06078800

a Abbreviation: N/A, not applicable.

b Data from ClinicalTrials.gov.

c Terminated/withdrawn clinical trials.

d Completed clinical trials.

## Highlights

After nearly 40 years of effort to develop a clinically relevant KRAS inhibitor, Kevan Shokat’s group identified the first mutant-selective inhibitor targeting KRAS^G12C^. This breakthrough reignited interest in KRAS as a drug target, culminating in FDA approval of the first mutant-selective KRAS inhibitors.

Despite the initial favorable response to KRAS^G12C^ inhibitors, resistance and disease progression are observed in most patients.

Numerous combinatorial therapies involving KRAS inhibitors are in clinical trials aiming to overcome therapy resistance. Additionally, other mutantspecific inhibitors (e.g., KRAS^G12D^), as well as pan-KRAS inhibitors or RAS (ON) multiselective inhibitors, are entering clinical trials and demonstrating promising results.

With the growing number of RAS inhibitors, patients with mutated KRAS are edging closer to an effective targeted therapy.

## Box 1. RAS signaling

RAS proteins belong to the family of small GTPases and function as molecular switches within the cell, toggling between an inactive GDP-bound state and an active GTP-bound state. The activation state of the RAS switch is regulated by active RTKs. These recruit guanine nucleotide exchange factors (GEFs), which facilitate the exchange of GDP to the more abundant GTP, thereby switching RAS on. To switch RAS off, GTPase-activating proteins (GAPs) bind to RAS and accelerate the hydrolysis of GTP to GDP [89]. Oncogenic mutations in RAS result in a predominantly GTP-loaded RAS.

In its active GTP-bound state, RAS recruits effector proteins containing a RAS-binding domain (RBD) to the plasma membrane. This RAS-mediated plasma membrane localization increases the local concentration of effector proteins, leading to their activation [147]. Key effector proteins include RAF, PI3K, RAL, RASSF, and TIAM, which activate distinct downstream pathways involved in processes such as proliferation, differentiation, survival, and endocytosis (Figure I).

## Outstanding questions

Although KRAS^G12C^ inhibitors demonstrate temporal efficacy in the clinical setting, ~50% of patients show no response to these therapies. What are the underlying mechanisms driving this primary resistance? Are there potential drug combinations that could overcome this resistance and improve the efficacy of treatment in these patients?

Can the drivers of primary resistance be identified and leveraged as potential biomarkers for predicting patient response? Additionally, are there specific biomarkers that can be used to anticipate the emergence of secondary resistance?

Besides primary resistance, the development of acquired resistance poses a significant challenge for KRAS^G12C^ inhibitors, with many underlying mechanisms yet to be fully understood. What are the other mechanisms of resistance that make patients unsuitable for continued KRAS^G12C^ therapy?

With numerous clinical trials exploring combinations of KRAS^G12C^ inhibitors with other therapies, which combinations are likely to provide the most significant therapeutic benefit? Will these combinations extend the progression-free period for patients?

RAS(ON) multiselective inhibitors are demonstrating promising preliminary clinical results, but the emergence of resistance remains a key challenge. A crucial question is whether the resistance mechanisms observed with KRAS^G12C^ inhibitors will predominantly drive resistance in multiselective RAS(ON) therapies or if novel mechanisms will emerge as the resistance drivers. In addition, will the combination strategies used with KRAS^G12C^ inhibitors be directly applicable to multiselective RAS(ON) therapies, or could more effective combination approaches be identified?

Considering that RAS is a small GTPase, can the insights gained from KRAS^G12C^ and RAS(ON) multiselective inhibitors be applied to other small GTPases such as RHO or RAB? Furthermore, could such inhibitors offer additional clinical benefits when targeting these GTPases?

## Resources

i
www.synapse.org/Synapse:syn52433756

ii
www.cbioportal.org/

iii
https://ir.revmed.com/news-releases/news-release-details/revolution-medicines-presents-initial-data-rmc-9805-monotherapy

iv
https://ir.revmed.com/news-releases/news-release-details/revolution-medicines-presents-updated-data-rmc-6236-monotherapy

v
www.amgen.com/newsroom/press-releases/2021/10/amgen-announces-new-lumakras-sotorasib-combination-data-from-phase-1b-codebreak-101-study-in-patients-with-kras-g12cmutated-cancers-at-aacrncieortc-2021

vi
https://investor.verastem.com/news-releases/news-release-details/verastem-oncology-announces-initial-results-ramp-203-trial/

## Glossary

Acquired resistance: resistance that develops over time in initially responsive cancer cells due to genetic or phenotypic changes that allow the cancer cells to survive despite inhibitor treatment.
Adaptive resistance: transient, reversible resistance mechanism in which cancer cells alter their signaling pathways or metabolism in response to treatment, allowing them to survive under inhibitor treatment.
Complete response: disappearance of all target lesions.
Disease control (DC): percentage of patients who show complete disappearance of the tumor or reduction in tumor size or no progression of tumor growth.
Epithelial–mesenchymal transition (EMT): process by which epithelial cells transform into mesenchymal cells, gaining migratory and invasive properties. EMT is associated with cancer metastasis and therapy resistance.
KRAS(OFF) inhibitors: targeted therapies that inhibit KRAS proteins in the inactive (GDP-bound) state, preventing their activation and subsequent oncogenic signaling. KRAS(OFF) inhibitors include sotorasib and adagrasib.
KRAS(ON) inhibitors: targeted therapies that inhibit KRAS proteins in the active (GTP-bound) state. Current examples include RMC-6291.
Objective response rate (ORR): percentage of patients who show at least a 30% reduction in the sum of lesion diameter or complete disappearance of the target lesions (PR and complete response, respectively).
Partial response (PR): at least a 30% reduction in the sum of target lesion diameter following treatment compared with the sum of lesion diameter at beginning of the treatment.
Primary resistance: inherent resistance of cancer cells to a therapy from the start of the treatment, due to pre-existing genetic or phenotypic characteristics that render the treatment ineffective.
Progression-free survival (PFS): period of time for which patients show no disease progression.
Progressive disease (PD): at least a 20% increase in the sum of the diameter of target lesions compared with the sum of diameter at beginning of the treatment or the appearance of one or more new lesions (even if the sum of target lesions shows a PR).
Receptor tyrosine kinase (RTK): cell surface receptors that facilitate tyrosine kinase phosphorylation.
Vertical inhibition: strategy that targets multiple points along the same signaling pathway to achieve a more comprehensive blockade of oncogenic signals, potentially overcoming resistance and improving outcomes.
